# Comparative study of major depressive symptoms among pregnant women by employment status

**DOI:** 10.1186/2193-1801-2-201

**Published:** 2013-04-30

**Authors:** Aïssatou Fall, Lise Goulet, Michel Vézina

**Affiliations:** École de Santé Publique de l’Université de Montréal (ÉSPUM), Québec, Canada; Institut national de santé publique du Québec, Québec, Canada; Département de médecine sociale et préventive, Université Laval, Québec, Canada

**Keywords:** Pregnancy, Employment status, Major depressive symptoms, Risk factors

## Abstract

The objectives of our study were to compare the prevalence of major depressive symptoms between subgroups of pregnant women: working women, women who had stopped working, housewives and students; and to identify risk factors for major depressive symptoms during pregnancy. The CES-D scale (Center for Epidemiological Studies Depression scale) was used to measure major depressive symptoms (CES-D score ≥23) in 5337 pregnant women interviewed at 24–26 weeks of pregnancy. Multivariate logistic regression models were developed to identify risk factors associated with major depressive symptoms. Prevalence of major depressive symptoms was 11.9% (11.0–12.8%) for all pregnant women. Working women had the lowest proportion of major depressive symptoms [7.6% (6.6–8.7%); n = 2514] compared to housewives [19.1% (16.5–21.8%); n = 893], women who had stopped working [14.4% (12.7–16.1%); n = 1665], and students [14.3% (10.3–19.1%); n = 265]. After adjusting for major risk factors, the association between pregnant women’s employment status and major depressive symptoms remained significant for women who had stopped working (OR: 1.61; 95% CI 1.26 to 2.04) and for housewives (OR: 1.46; 95% CI 1.10 to 1.94), but not for students (OR: 1.37; 95% CI 0.87 to 2.16). In multivariate analyses, low education, low social support outside of work, having experienced acute stressful events, lack of money for basic needs, experiencing marital strain, having a chronic health problem, country of birth, and smoking were significantly associated with major depressive symptoms. Health professionals should consider the employment status of pregnant women when they evaluate risk profiles. Prevention, detection and intervention measures are needed to reduce the prevalence of prenatal depression.

## Background

During pregnancy anticipated changes in lifestyle and the thought of becoming a parent are causes of stress which, for some women, can affect physical, psychological and emotional well-being. Recent research suggests that prenatal depression is as prevalent as postpartum depression (Evans et al. 2001). The estimated prevalence rates of depression (95% CIs) are 7.4% (2.2–12.6%), 12.8% (10.7–14.8%), and 12.0% (7.4–16.7%) for the first, second, and third trimesters, respectively (Bennett et al. 2004). Several prior studies have shown that women with elevated depressive symptoms have increased risks of poor pregnancy outcomes (Orr et al. 2007). Moreover, prenatal depression is the main risk factor for postnatal depression (Bennett et al. 2004). In fact, about 20% of postnatal depressions are extensions or recurrences of depression that occurred during pregnancy (Wisner et al. 2004).

Few studies have looked at the risk factors for depressive symptoms during pregnancy (Rich-Edwards et al. 2006; Lancaster et al. 2010; Witt et al. 2010). Maternal anxiety, life stress, history of depression, lack of social support, unintended pregnancy, domestic violence, lower income, lower education, smoking, single status, and poor relationship quality were associated with increased risks of depressive symptoms during pregnancy (Lancaster et al. 2010). None has focused on the effects of employment status on women’s mental health during pregnancy, although we know that increasingly more women of childbearing age form a significant proportion of the workforce and that prevalences of depression and anxiety are higher during active life and reproductive years (20–40 years) (Stansfeld et al. 1999). Furthermore, many women combine several roles during pregnancy, such as working or seeking employment, going to school or managing the home (Plaisier et al. 2008).

Work can be beneficial for mental health but it can also be detrimental (Stansfeld et al. 1999; Karasek & Theorell 1990; Iacovides et al. 2003). Having a job keeps a person busy, raises socioeconomic status and provides opportunities for self-fulfilment (Sieber 1974). On the other hand, some work characteristics can be sources of high strain job that can lead to mental health problems (Karasek & Theorell 1990; Iacovides et al. 2003; Sieber 1974; Virtanen et al. 2007; Plaisier et al. 2007). Overall, it has been noted that having a job has a positive effect on women’s health, and that the increasing participation of women in the workforce over the last decades has not negatively affected their health but rather has improved it (Hall 1992). Working women are in better physical and psychological health than housewives (Hall 1992; Baruch & Barnett 1986; Matthews et al. 2001), and have lower levels of morbidity and mortality than unemployed women and housewives (Silman 1987). When work conditions, demographic and family characteristics are taken into account, housewives show more depressive symptoms than working married women (Lennon 1994). Women’s levels of distress, of acute and chronic stress are particularly high among unemployed single mothers (Steward et al. 2003). However, the positive association between employment and women’s health status may also reflect the selection of women being able to work, compared to those out of the labor force for health or family reasons (Vermeulen & Mustard 2000). Only a few studies have not found significant differences in mental health status between women who work and those who do not (Warr & Parry 1982; Waldron et al. 1982; Waldron 1991).

The aim of this study was to examine the association between employment status during pregnancy and mental health. More specifically, we compared the prevalence of major depressive symptoms between different subgroups of pregnant women: working women, women who had stopped working, housewives and students. We also identified the risk factors associated with major depressive symptoms.

## Methodology

Analyses were carried out using data from the Montreal Prematurity Study (Kramer et al. 2001), a prospective cohort study of spontaneous preterm birth among 5337 women who delivered from May 1999 to April 2004 in four large maternity hospitals affiliated with McGill University and the Université de Montréal. These hospitals serve a wide socioeconomic spectrum, and are representative of French- and English-speaking populations across the Island of Montréal, Canada. The project was approved by the ethics committees at all four participating hospitals.

Women were recruited when they presented for routine ultrasound examinations (16–20 weeks), for prenatal blood drawing (8–12 weeks) or in the hospital’s prenatal clinic (before 24 weeks). Eligibility criteria were as follows: age ≥18 years at expected date of delivery, singleton gestation, and fluency in French or English. Women presenting a severe chronic illness (other than hypertension, asthma, or diabetes) or a condition that increased the risk of preterm birth (placenta prævia, history of incompetent cervix, foetal anomaly) were excluded from the study. Women who accepted to participate in the study were requested to return to a special research clinic at 24–26 weeks of gestation for an interview and examination, performed by a research nurse.

The following variables were analyzed:

### Employment status

Women were categorized in four groups:

- *Working women*: women who were still working at time of prenatal interview
- *Women who had stopped working*: women who had stopped working since the beginning of pregnancy for various reasons (e.g. preventive withdrawal from work, end of contract, fired, quit, sick leave, other)
- *Housewives*: women who had not had a paid job outside the home since the beginning of pregnancy
- *Students*: women who had been attending an educational institution or university since the beginning of pregnancy and did not have a paid job

### Major depressive symptoms

The 20-item version of the CES-D scale (Center for Epidemiological Studies Depression Scale), initially designed to measure depressive symptoms in the community, was used in our sample to identify the presence and assess the severity of depressive symptoms (Radloff 1977). It allows measurement of depressed mood and symptoms over the past seven days (e.g. sadness, hopelessness, fatigue, crying, sleep disturbances and loss of appetite). The scale has excellent internal consistency, with Cronbach’s alpha ranging from 0.85 (general population) to 0.90 (psychiatric patients), and adequate test-retest reliability (0.54), for a scale designed to be sensitive to adverse changes in a respondent’s environment (Radloff 1977). CES-D scores range from zero to 60 and correlate well with clinical diagnoses of major depressive disorder (Radloff 1977).

We used the cutoff of CES-D score ≥ 23 proposed by Radloff and Locke (Radloff & Locke 1986) to indicate the presence of major depressive symptoms. Considering that certain symptoms of depression overlap with symptoms of pregnancy (e.g. appetite change and fatigue), women who rank in the upper 10 percent of CES-D scores would have larger number and frequency of symptoms and meet the criteria for clinical depression.

### Covariates

#### Demographic and socioeconomic characteristics, lifestyle habits, chronic health problems

The following characteristics were measured at the time of the interview: mother’s age; country of birth (Canada, other countries); parity (primiparous, multiparous); single-parent family (yes/no); highest level of education; annual household income; smoking during pregnancy (non-smoker, occasionally/regularly); alcohol consumption during pregnancy (no alcohol, ≤2 times/month, several times/week) and chronic health problems during pregnancy (none, at least one).

#### Stressful life events

The abbreviated version (16 items) of Lobel’s Prenatal Life Events Scale (PLES) was used (Lobel 1997). Recent upsetting events that had occurred since the beginning of pregnancy included moving, unwanted cohabitation, theft, natural catastrophes, racial discrimination, accidents, job loss, separation or divorce, woman herself or family member seriously ill, and death of a spouse or loved one. Life events were summed and a three-category variable was created (none, one or two, more than two life events).

#### Lack of money for basic needs

We used a subscale from the Daily Hassles Scale (Kanner et al. 1981), the “lack of money for essential needs scale”, to measure how often and to what extent the woman had lacked money for basic needs such as food, heating, electricity, bills, rent, medications and other necessities since the beginning of pregnancy. A validation study showed adequate internal consistency (Cronbach’s α = 0.79) (Ehounoux et al. 2009). Responses to each item were summed and the variable was dichotomized as yes (for those who have lacked money for three or more essential needs indicative of poverty) or no (all others).

#### Marital strain

Pearlin and Schooler’s Marital Strain Scale (9 items) was used to assess chronic stress with the woman’s partner (Pearlin & Schooler 1978). We used quartiles of distribution to construct a four-category variable, keeping women who did not have a partner into a separate category to avoid their exclusion: no marital strain (lowest quartile), low or moderate marital strain (second and third quartiles), high marital strain (highest quartile), no partner.

#### Social support outside work

Social support outside work was determined using the Arizona Social Support Interview Schedule’s availability of help from the social network (Barrera 1981). The scale measures not getting help when needed in five functions of support: instrumental, emotional, informative, normative, and companionship. Cronbach’s α varies from 0.70 to 0.78 (Barrera 1981) for these five functions. Social support was dichotomized as absent (those with unmet needs in one or more of the five areas) or present (those without needs and those whose needs were met).

### Data analyses

First we compared the distribution of major depressive symptoms and covariates by employment status using Chi square tests for categorical variables and one-way ANOVA for continuous variables.

Then, we calculated the prevalence of major depressive symptoms according to employment status (and 95% CI) and each covariate, and assessed the association between these variables and major depressive symptoms using logistic regression (unadjusted OR and 95% CI).

Finally we constructed multivariate logistic models to compare women by employment status during pregnancy.

To select potential confounders and mediators, we performed a series of analyses. We started to calculate the overall effect of employment status (Model 1) and to evaluate the individual effects of all covariate, these factors were added separately to Model 1. For each adjustment, the percentage change in OR for employment status with an increased risk for major depressive symptoms was calculated (100 X [OR _Model 1_ – OR _+covariate_]/[OR _Model 1_ - 1]) (MacKinnon et al. 2000). Only factors that individually produced at least a 10% change (Rothman & Greenland 1998) in the OR for the employment status were kept for the multivariate analysis.

We then assessed the effect of explanatory variables by staggered entry of demographic variables (Model 2) followed by socioeconomic variables, lifestyle habits and stressors (Model 3).

Goodness of fit was assessed by the Hosmer and Lemeshow test (xHosmer & Lemeshow 1989). Moreover, ROC (Receiver Operating Characteristic) curves were plotted and areas under the ROC curve were analyzed and tested (p < 0.05) for goodness of fit of the different models.

All reported p-values were 2-sided and p-value <0.05 was considered statistically significant.

Analyses were performed using IBM® SPSS® Statistics 20 for Windows.

## Results

The characteristics of our study population according to employment status are presented in Table 1. Of the 5337 pregnant women, 78.3% worked during pregnancy (47.1% were still working at the time of interview and 31.2% had stopped working), 16.7% were housewives, and 5.0% students. Women were at an average of 25.0 ± 0.6 weeks of gestation (range 24 to 26 weeks) and their average age was 29.5 ± 5.35 years (range: 18 to 49 years).

**Table 1:** **Demographic, socioeconomic and psychological characteristics of pregnant women, by employment status**

	Pregnant women’s employment status				
	Total	Working women	Women who had stopped working	Housewives	Students
	N = 5337	N = 2514 (47.1%)	N = 1665 (31.2%)	N = 893 (16.7%)	N = 265 (5.0%)
**Demographic variables**					
**Mother’s age (years)** (n = 5334)					
< 25 years (%)	19.8	**11.7** ^**a b c**^	**26.7**	**28.1** ^**d e**^	**25.7**
25-34 years (%)	61.8	**65.9**	**59.6**	**54.1**	**63.0**
≥ 35 years (%)	18.4	**22.4**	**13.7**	**17.8**	**11.3**
**Country of birth** (n = 5334)					
Canada (%)	71.8	**75.8** ^**b c**^	**75.7**	**63.6** ^**d e**^	**37.7** ^**f**^
Other countries (%)	28.2	**24.2**	**24.3**	**36.4**	**62.3**
**Parity** (n = 5328)					
Primiparous (%)	58.5	**63.4** ^**b c**^	**63.5**	**36.3** ^**d e**^	**56.6** ^**f**^
Multiparous (%)	41.5	**36.6**	**36.5**	**63.7**	**43.4**
**Single-parent family** (n = 5313)					
No (%)	89.5	**94.0** ^**a b c**^	**88.8**	**79.8** ^**d**^	**84.5** ^**f**^
Yes (%)	10.5	**6.0**	**11.2**	**20.2**	**15.5**
**Level of education** (n = 5337)					
Partial high school (%)	15.6	**6.9** ^**a b c**^	**17.3**	**37.8** ^**d e**^	**12.8** ^**f**^
Partial college (%)	16.9	**13.6**	**21.4**	**17.7**	**17.8**
College completed/Partial university (%)	29.5	**29.9**	**33.9**	**21.1**	**26.0**
University degree (%)	37.9	**49.5**	**27.4**	**23.4**	**43.4**
**Socioeconomic variables, lifestyle habits and stressors**					
**Household income** (n = 5337)					
<$15 000	10.5	**4.3** ^**a b c**^	**9.8**	**25.3** ^**d**^	**23.8** ^**f**^
≥ $15,000 and < $30,000	13.8	**10.4**	**16.9**	**15.8**	**18.9**
≥ $30,000 and < $50,000	20.3	**19.3**	**25.3**	**14.3**	**17.7**
≥ $50,000 and < $80,000	24.1	**30.5**	**23.1**	**12.2**	**8.7**
≥ $80,000	18.7	**27.9**	**12.3**	**8.5**	**6.0**
Not revealed	12.7	**7.6**	**12.6**	**23.9**	**24.9**
**Reported a lack of money** (n = 5302)					
No (%)	74.1	**78.6** ^**a b c**^	**71.8**	**66.4** ^**d**^	**71.6**
Yes (%)	25.9	**21.4**	**28.2**	**33.6**	**28.4**
**Smoking during pregnancy** (n = 5287)					
Non-smoker (%)	84.1	**89.8** ^**a b c**^	**79.7**	**76.0** ^**d e**^	**84.8**
Occasionally/Regularly (%)	15.9	**10.2**	**20.3**	**24.0**	**15.2**
**Alcohol consumption during pregnancy** (n = 5333)					
No alcohol (%)	46.9	**41** ^**a b c**^	**46.5**	**60.7** ^**d**^	**59.7** ^**f**^
≤ 2 times/month (%)	46.7	**50.8**	**48.8**	**35.3**	**32.8**
Several times/week	6.4	**8.2**	**4.7**	**4.0**	**7.5**
**Chronic health problem** (n = 5337)					
None (%)	84.4	**86.6** ^**a b**^	**82.8**	**80.2** ^**e**^	**86.8**
At least one (%)	15.6	**13.4**	**17.2**	**19.8**	**13.2**
**Stressful life events** (n = 5273)					
None (%)	23.3	**28.0** ^**a b c**^	**19.0**	**18.8**	**22.1**
One or two (%)	51.6	**52.3**	**51.5**	**49.4**	**51.9**
More than two (%)	25.1	**19.7**	**29.5**	**31.8**	**26.0**
**Social support outside work** (n = 5337)					
Present (%)	88.4	**90.8** ^**b c**^	**89.5**	**81.4** ^**d**^	**81.9** ^**f**^
Absent (%)	11.6	**9.2**	**10.5**	**18.6**	**18.1**
**Marital strain** (n = 5191)					
No marital strain	28.4	**29.9** ^**a b c**^	**28.7**	**24.8** ^**d**^	**23.7** ^**f**^
Low or moderate marital strain	39.5	**41.9**	**39.7**	**33.2**	**36.7**
High marital strain	26.3	**25.4**	**26.0**	**28.6**	**29.8**
No partner	5.8	**2.8**	**5.6**	**13.4**	**9.8**
**Depressive symptoms**					
**CES-D score** (n = 5169)					
Mean (SD)	11.3 (8.64)	**10.45 (7.53)** ^**a b c**^	**12.79 (8.85)**	**14.20 (10.19)** ^**d**^	**13.40 (9.27)**
**Major depressive symptoms** (n = 5169)					
% CES-D ≥ 23 (95% CI)	11.9 (11.0-12.8)	**7.6 (6.6-8.7)** ^**a b c**^	**14.4 (12.7-16.1)**	**19.1(16.5-21.8)** ^**d**^	**14.3 (10.3-19.1)**

^**a**^ Significant differences in proportions or means between **working women** and **women who had stopped working,** P < 0.05.

^**b**^ Significant differences in proportions or means between **working women** and **housewives**, P < 0.05.

^**c**^ Significant differences in proportions or means between **working women** and **students,** P < 0.05.

^**d**^ Significant differences in proportions or means between **housewives** and **women who had stopped working**, P < 0.05.

^**e**^ Significant differences in proportions or means between **housewives** and **students**, P < 0.05.

^**f**^ Significant differences in proportions or means between **students and women and who had stopped working**, P < 0.05.

Compared to other women, working women (n = 2514) were older, had the highest education and socio-economic levels, and were more likely to be in a relationship and to drink alcohol; they were less likely to smoke and to report lack of money, stressful life events and marital strain.

Women who had stopped working (n = 1665) had mixed profiles. They were comparable to working women for country of birth, parity and social support outside work, but more similar to housewives for chronic health problems and stressful life events. The reasons why women had stopped working were preventive withdrawal from work because of dangerous working conditions (61.4%), end of contract/fired/quit (22.3%), sick leave (11,4%), other (4,9%).

Housewives (n = 893) showed high frequencies of multiparity, single-parent family, low education, low income, lack of money for basic needs, chronic health problems, stressful life events and lack of social support outside of work.

Students (n = 265) were mainly foreign-born women (62.3%) with high levels of education (43.4% had university degrees). They had low income, reported lack of social support outside work but had good lifestyles and health.

Scores on the CES-D scale ranged from 0 to 54, with an average score of 11.3 ±8.6 and a median of 10. (Table 1). Scores in the upper 10th percentile were from 23 through 54 (n = 616).

Prevalence of major depressive symptoms was 11.9% (11.0–12.8%) for all pregnant women. Working women had better mental health than other subgroups of pregnant women. They had the lowest proportion of major depressive symptoms [7.6% (6.6–8.7%)] compared to housewives [19.1% (16.5–21.8%)], women who had stopped working [14.4% (12.7–16.1%)], and students [14.3% (10.3–19.1%)]. The difference was also significant between housewives and pregnant women who had stopped working (p < 0.002).

Table 2 shows the associations between covariates and major depressive symptoms in bivariate analyses. In our sample, prevalence of major depressive symptoms was higher among women who were single parents (29.1%), had partial high school (24.1%), low household income (25.7%), reported stressful life events (22.3%), lacked social support outside work (40.7%) or declared high marital strain (22.2%). All variables except alcohol consumption were significantly associated with the outcome.

**Table 2:** **Variables associated with major depressive symptoms (CES-D score ≥ 23)**

Variables	Major depressive symptoms	
	CES-D score ≥ 23	
	%	Unadjusted odds ratio (95% CI)
**Employment status** (n = 5169)		
Working women	7.6	1.00
Women who had stopped working	14.4	2.04 (1.66-2.50) ***
Housewives	19.1	2.87 (2.28-3.60) ***
Students	14.3	2.02 (1.37-2.98) ***
**Mother’s age (years)** (n = 5166)		
< 25 years	19.2	1.00
25-34 years	10.3	0.48 (0.39-0.58) ***
≥ 35 years	9.7	0.45 (0.34-0.58) ***
**Country of birth** (n = 5166)		
Canada	10.4	1.00
Other countries	15.9	1.65 (1.38-1.98) ***
**Parity** (n = 5161)		
Primiparous	10.8	1.00
Multiparous	13.5	1.30 (1.09-1.54) **
**Single-parent family** (n = 5147)		
No	9.9	1.00
Yes	29.1	3.76 (3.05-4.65) ***
**Level of education** (n = 5169)		
Partial high school	24.1	1.00
Partial college	14.4	0.52 (0.41-0.67) ***
College completed/Partial university	11.0	0.38 (0.31-0.48) ***
University degree	6.5	0.22 (0.17-0.28) ***
**Household income** (n = 5169)		
<$15 000	25.7	1.00
≥ $15,000 and < $30,000	16.1	0.55 (0.41-0.73) ***
≥ $30,000 and < $50,000	10.7	0.34 (0.26-0.45) ***
≥ $50,000 and < $80,000	6.0	0.18 (0.13-0.25) ***
≥ $80,000	4.5	0.13 (0.09-0.19) ***
Not revealed	20.8	0.75 (0.57-0.99) *
**Reported a lack of money (** n = 5136)		
No	9.0	1.00
Yes	20.0	2.51 (2.11-2.99) ***
**Smoking during pregnancy** (n = 5121)		
Non-smoker	10.4	1.00
Occasionally/Regularly	20.1	2.17 (1.78-2.65) ***
**Alcohol consumption during pregnancy** (n = 5165)		
No alcohol	12.8	1.00
≤ 2 times/month	11.2	0.85 (0.71-1.01)
Several times/week	10.9	0.82 (0.57-1.19)
**Chronic health problem** (n = 5169)		
None	10.6	1.00
At least one	18.9	1.95 (1.60-2.39) ***
**Stressful life events** (n = 5114)		
None	5.8	1.00
One or two	9.6	1.72 (1.31-2.27) ***
More than two	22.3	4.70 (3.56-6.20) ***
**Social support outside work** (n = 5169)		
Present	8.2	1.00
Absent	40.7	7.66 (6.30-9.31) ***
**Marital strain** (n = 5061)		
No marital strain	3.7	1.00
Low or moderate marital strain	7.0	1.95 (1.41-2.69) ***
High marital strain	22.2	7.37 (5.46-9.96) ***
No partner	36.3	14.74(10.23-21.22) ***

*p <0 **.05.** **p <0 .01. ***p < 0.001.

CES-D: Center for Epidemiologic Studies Depression Scale.

In multivariate analyses, after adjusting for all variables (Tables 3: Model 3), the association between major depressive symptoms and pregnant women’s employment status remained significant for women who had stopped working (adjusted OR: 1.61, 95% CI 1.26 to 2.04) and for housewives (adjusted OR: 1.46, 95% CI 1.10 to 1.94) but not for students (adjusted OR: 1.37, 95% CI 0.87 to 2.16). Adjustments for demographic characteristics brought the most significant changes in ORs for women’s employment status, figures which barely changed after adjustment for the other covariates.

**Table 3:** **Logistic regression models for major depressive symptoms (CES-D score ≥ 23)**

Variables	Models		
	Model 1 Unadjusted OR (95% CI)	Model 2 Adjusted OR (95% CI)	Model 3 Adjusted OR (95% CI)
**Employment status**			
Working women (Ref.)	1.00	1.00	1.00
Women who had stopped working	2.04 (1.66-2.50) ***	1.60 (1.30-1.98) ***	1.61 (1.26-2.04) ***
Housewives	2.87 (2.28-3.60) ***	1.69 (1.32-2.17) ***	1.46 (1.10-1.94) **
Students	2.02 (1.37-2.98) ***	1.39 (0.93-2.08)	1.37 (0.87-2.16)
**Demographic variables**			
**Age group (years)**			
< 25 years (Ref.)		1.00	1.00
25-34 years		0.81 (0.65-1.01)	0.91 (0.71-1.17)
≥ 35 years		0.77 (0.57-1.05)	0.77 (0.55-1.09)
**Country of birth**			
Canada (Ref)		1.00	1.00
Other countries		1.70 (1.40-2.05)***	1.25 (1.01-1.57)*
**Single-parent family**			
No (Ref)		1.00	
Yes		2.43 (1.93-3.05)***	1.12 (0.76-1.64)
**Level of education**			
Partial high school (Ref.)		1.00	1.00
Partial college		0.63 (0.49-0.83) **	0.70 (0.52-0.94) *
College completed/Partial university		0.55 (0.43-0.71) ***	0.71 (0.53-0.96) *
University degree		0.35 (0.26-0.46) ***	0.47 (0.34-0.66) ***
**Socioeconomic variables, life style habits and stressors**			
**Reported a lack of money**			
No (Ref.)			1.00
Yes			1.30 (1.06-1.61) *
**Smoking during pregnancy**			
Non-smoker (Ref)			1.00
Occasionally/Regularly			1.44 (1.13-1.84) **
**Chronic health problem**			
None (Ref.)			1.00
At least one			1.31 (1.03-1.66) *
**Stressful life events**			
None (Ref.)			1.00
One or two			1.25 (0.92-1.70)
More than two			2.20 (1.60-3.02) ***
**Social support outside work**			
Present (Ref.)			1.00
Absent			4.47 (3.55-5.63) ***
**Marital strain**			
No marital strain (Ref.)			1.00
Low or moderate marital strain			1.54 (1.10-2.16) *
High marital strain			4.27 (3.11-5.88) ***
No partner			5.07 (2.98-8.63) ***
**Model results**			
**Overall percentage of correct classification**	88.1%	88.2%	89.3%
**−2 log likelihood**	3680.82	3468.58	2780.16
**Model Chi-square**	95.36	293.04	758.13
Degree of freedom	3	10	19
P-value	**<0.0001**	**<0.0001**	**<0.0001**
**ROC curve**			
Area under the curve (95% CI)	0.611 (0.588-0.635)	0.703 (0.681-0.724)	0.818 (0.800-0.836)
P-value	**<0.0001**	**<0.0001**	**<0.0001**

Model 1: Employment status of pregnant women.

Model 2: Model 1 + Demographic variables.

Model 3: Model 2 + Socioeconomic variables, lifestyle habits and stressors.

*p <0 **.05.** **p <0 .01. ***p < 0.001.

The CES-D score ≥ 23 was strongly associated with low social support outside work (adjusted OR: 4.47, 95% CI 3.55 to 5.63), experiencing high marital strain (adjusted OR: 4.27, 95% CI 3.11 to 5.88) and having experienced acute stressful events (adjusted OR: 2.20, 95% CI 1.60 to 3.02). Other risk factors associated with major depressive symptoms were smoking during pregnancy, chronic health problems, lack of money for basic needs, and country of birth. Having a high level of education appeared to be a protective factor.

Post hoc multivariate logistic regression analyses were performed separately for each subgroup of pregnant women. The analyses revealed that low social support outside work was the strongest risk factor for major depressive symptoms and that having a high level of education remained a protective factor regardless of the subgroup of pregnant women.

## Discussion

At 24 to 26 weeks of pregnancy, prevalence of major depressive symptoms was 11.9% (11.0–12.8%) for all pregnant women and varied significantly with women’s employment status. Based on the results of two meta-analyses, the average prevalence rate of prenatal depression is estimated to be approximately 12% (Bennett et al. 2004; Gavin et al. 2005), while prevalence may vary up to 18% (Marcus et al. 2003) according to mode of assessment and socioeconomic conditions. Orr et al. have shown prevalences of major depressive symptoms (CES-D score ≥ 23) ranging from 16.2% to 27.5% for multiracial pregnant women (Orr et al. 2006). Prevalence results for the different subgroups of pregnant women in our study fall within these intervals, with CES-D scores ≥ 23 ranging from 7.6% (6.6–8.7%) for working women to 19.1% (16.5–21.8%) for housewives.

Our analyses reveal notable variations in the profiles of these subgroups of women. It is interesting to note that after adjusting for demographic and socioeconomic characteristics, lifestyle habits, and acute and chronic stressors, women’s employment status remained independently associated with major depressive symptoms. Women who had stopped working and housewives had approximately 47% and 61% greater prevalences of major depressive symptoms (CES-D score ≥23) than working women.

The better mental health of pregnant working women in our sample was not a surprise; this phenomenon is well documented in epidemiology and is known as the “Healthy Worker Effect” (Vinni & Hakama 1980). Indeed it is a well-established and undisputed phenomenon that employers select the healthiest people for jobs and people who are not employed are physically and mentally less healthy than people who are employed, due to a selection bias (Waldron 1991; Repetti et al. 1989). However, this phenomenon is complex and involves factors other than selection bias. One possible explanation for this finding is that working women are also more educated and may have a better sense of how to lead a healthy lifestyle.

Housewives appeared to be at highest risk of prenatal depression considering their frequency exposure to known risk factors such as low level of education, immigrant status, low income, stressful life events, lack of social support and marital strain which, in our study, were once again independently associated with prenatal depressive symptoms (Bennett et al. 2004; Rich-Edwards et al. 2006; Lancaster et al. 2010; Marcus et al. 2003; Westdahl et al. 2007; Zelkowitz et al. 2004). Even after adjusting for these factors, housewives had a greater prevalence of major depressive symptoms than working women. In our study, pregnant women’s personality traits (e.g. low self-esteem, anxiety), and personal and family histories of depression were not documented, even though these factors have been found to be strongly associated with depression in pregnant women (Bunevicius et al. 2009).

Women who had stopped working during pregnancy were less educated than working women and experienced more economic problem and major depressive symptoms. Worsening mental health might contribute to the decision to stop working during pregnancy (Matsuzaki et al. 2011). Furthermore, working conditions such as psychosocial work demands, and change in employment status can influence the incidence of severe depressive symptoms. Further research is needed to gain a better understanding of why pregnant women who stop working have high rates of major depressive symptoms.

Students had profiles that put them at higher risk of prenatal depression when compared to working women. In multivariate analyses, students’ demographic characteristics explained much of the difference in prevalence rates; OR were no longer statistically significant after adjusting for all variables, probably due to a lack of power.

Our study has strengths. We analyzed cross-sectional data from a large prospective multicentre cohort study of a sociodemographically diverse population recruited in four large Montreal maternity hospitals (Kramer et al. 2001). Another strength of our study is the detailed information we had on risk factors such as socioeconomic characteristics, lifestyles habits and stressors.

It also has several limitations. Pregnant women recruited in this cohort were more educated than women who gave birth in hospitals located in the Montréal census metropolitan area (Kramer et al 2009). This could lead to an underestimation of prevalence. Moreover, major depressive symptoms were self-reported and not based on clinical diagnosis. It is possible that education level, and cultural and/or economic factors may have contributed to over- or under-reporting depressive symptoms (Rich-Edwards et al. 2006). Furthermore, these symptoms were measured using the CES-D scale with a cutoff score of ≥23 rather than the Structured Clinical Interview for Depression (SCID) (Spitzer et al. 1992) who is considered to be the “gold standard” for the research diagnosis of depression (Spitzer et al. 1992; Marcus 2009).

Perinatal depression is multifactorial and constitutes a major public health problem. Routine depression screening for all pregnant women is imperative during each trimester. In addition, optimising earlier identification of major risk factors such as low social support, stressful life events, high marital strain, and low income for certain subgroups of pregnant women could be beneficial for detection and treatment of prenatal depression. Health professionals should consider the employment status of pregnant women when they evaluate risk profiles. Preventive measures are needed to reduce the prevalence of prenatal depression due to its effect not only on the mothers (recurrent depression), but also on the children who may be at greater risk of prematurity and low birth weight (Marcus et al. 2003; Marcus 2009). Hence, there is a need to implement primary (information, education and social support), secondary (screening and detection) and tertiary (intervention) preventive measures centred on the most vulnerable groups.

## Ethics approval

Approval for this study was obtained from all obstetricians performing deliveries at the four study hospitals and by the ethics committees at all four hospitals affiliated with McGill University and l’Université de Montréal: the Royal Victoria Hospital, Jewish General Hospital, Centre hospitalier de l’Université de Montréal, and Hôpital Maisonneuve-Rosemont.
